# Comparative Transcriptomic Analysis of WSSV-Challenged *Penaeus vannamei* with Variable Resistance Levels

**DOI:** 10.3390/ijms25094961

**Published:** 2024-05-02

**Authors:** Xupeng Li, Qian Xue, Sheng Luan, Kun Luo, Jie Kong, Xianhong Meng

**Affiliations:** 1 State Key Laboratory of Mariculture Biobreeding and Sustainable Goods, Yellow Sea Fisheries Research Institute, Chinese Academy of Fishery Sciences, Qingdao 266071, China; 2 Laboratory for Marine Fisheries Science and Food Production Processes, Qingdao Marine Science and Technology Center, Qingdao 266237, China

**Keywords:** WSSV, *Penaeus vannamei*, immunity, family, resistant ability, viral load, transcriptome

## Abstract

The Pacific white shrimp, *Penaeus vannamei*, is highly susceptible to white spot syndrome virus (WSSV). Our study explored the transcriptomic responses of *P*. *vannamei* from resistant and susceptible families, uncovering distinct expression patterns after WSSV infection. The analysis revealed a higher number of differentially expressed genes (DEGs) in the susceptible family following WSSV infection compared to the resistant family, when both were evaluated against their respective control groups, indicating that the host resistance of the family line influences the transcriptome. The results also showed that subsequent to an identical duration following WSSV infection, there were more DEGs in *P*. *vannamei* with a high viral load than in those with a low viral load. To identify common transcriptomic responses, we profiled DEGs across families at 96 and 228 h post-infection (hpi). The analysis yielded 64 up-regulated and 37 down-regulated DEGs at 96 hpi, with 33 up-regulated and 34 down-regulated DEGs at 228 hpi, showcasing the dynamics of the transcriptomic response over time. Real-time RT-PCR assays confirmed significant DEG expression changes post-infection. Our results offer new insights into shrimp’s molecular defense mechanisms against WSSV.

## 1. Introduction

The Pacific white shrimp *Penaeus vannamei*, also known as the whiteleg shrimp, is a species of marine crustacean widely cultured worldwide due to its quick growth rate, high yield, and adaptability. However, white spot disease (WSD) presents a major challenge in shrimp aquaculture, causing substantial economic losses [1]. The white spot syndrome virus (WSSV), the etiological agent behind WSD, is capable of infecting nearly all commercial shrimp species and inducing an almost 100% mortality rate rapidly post-infection [2]. WSSV is an enveloped double-stranded DNA virus, classified within the genus *Whispovirus* of the family Nimaviridae [3]. WSSV exhibits a wide host range, capable of infecting shrimp, crabs, lobsters, crayfish, and copepods [4]. Additionally, WSSV is characterized by its ability to be transmitted vertically [5], adding to the challenges in managing and controlling the spread of this virus. These features significantly complicate the prevention and control of WSSV, emphasizing the need for comprehensive strategies in the aquaculture industry.

Although extensive research has been carried out on the molecular mechanisms of shrimp resistance to WSSV, the specific mechanisms of shrimp resistance to WSSV have not yet been accurately elucidated. WSSV remains one of the major threats to the global shrimp industry. Transcriptome sequencing is a powerful tool currently used to decipher the mechanisms of disease resistance. There is currently a wealth of reports on transcriptomes related to shrimp resistance against WSSV. In the year 2013, 454 pyrosequencing-based RNA-Seq technology was employed to analyze the transcriptome of *P*. *vannamei* hepatopancreas post WSSV infection, revealing the differential expression of genes involved in apoptosis, MAPK signaling, TLR signaling, Wnt signaling, and antigen processing and presentation pathways [6]. In the year 2019, using RNA-seq transcriptome of *P*. *vannamei* gill at 1.5, 18 and 56 h post-WSSV infection was analyzed. It was found that gene expression changes were dynamic and varied with time, highlighting the complexity of the host–pathogen interaction [7]. In the year 2022, Illumina HiSeq X Ten (Illumina, San Diego, CA) Sequencing technology was utilized to examine the transcriptome of *P*. *vannamei* hepatopancreas at 2, 36, and 48 h after WSSV infection. This analysis revealed the presence of immune, apoptosis, cytoskeleton, and antioxidant-related genes, indicating their involvement in the response to viral infection. Additionally, genes associated with phagocytosis, detoxification, blood coagulation, and energy metabolism were also identified, suggesting their potential roles in the host–virus interaction [8].

However, we were unable to find studies that compare the transcriptomic differences between individual *P*. *vannamei* from families with different resistance traits to WSSV, specifically at the same infection times but with varying levels of viral load. To investigate the genetic basis of WSSV resistance, we carried out a WSSV challenge on 59 *P*. *vannamei* families. From the outcomes, we identified and selected two families that demonstrated marked contrasts in WSSV resistance—one with high resistance and one with low. We subjected both families to a subsequent challenge test. At a consistent post-infection time point, individuals from each family were chosen based on their distinct viral load—some with high and others with low. Transcriptome analysis was conducted on the hepatopancreas, a key organ in shrimp innate immunity, of these selected shrimps.

## 2. Results

### 2.1. Quality of the Sequencing Data

In the transcriptome sequencing of 50 samples, we obtained an average of 40,714,253 raw reads and 6,107,137,938 raw bases per sample. After quality filtering, the averages became 40,442,320 clean reads and 6,016,292,948 clean bases. The clean bases achieved average Q20 and Q30 scores of 97.97% and 94.34%, with a mean GC content of 50.12%. More details are presented in Supplementary Table S1. The raw data are available at the NCBI SRA database (PRJNA1101112).

### 2.2. Identification of DEGs

**Figure 1:**
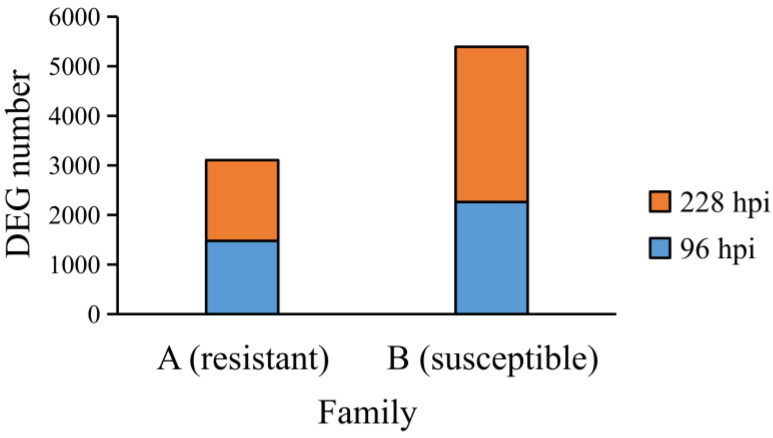
The DEG number in resistant family and susceptible family. The susceptible family exhibited a higher number of DEGs compared to the resistant family. (**A**) resistant family. (**B**) susceptible family.

**Figure 2:**
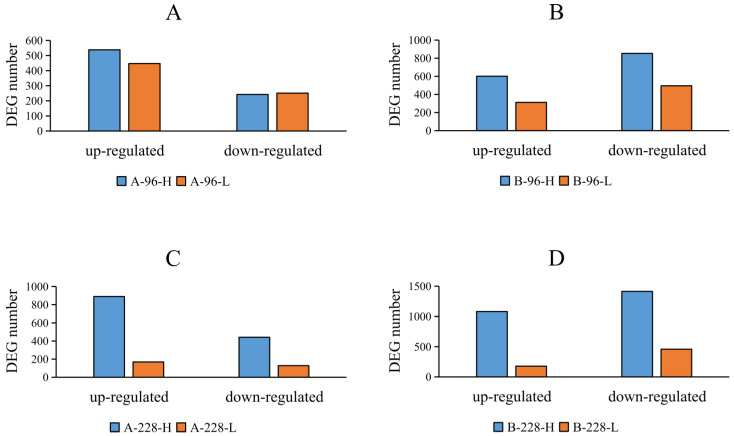
The number of up-regulated and down-regulated DEGs in different groups. The groups with high viral load exhibited a higher number of DEGs compared to those with low viral load. This trend was particularly evident in the samples collected at 228 hpi. (**A**) A-96-H and A-96-L groups. (**B**) B-96-H and B-96-L groups. (**C**) A-228-H and A-228-L groups. (**D**) B-228-H and B-228-L groups.

**Figure 3:**
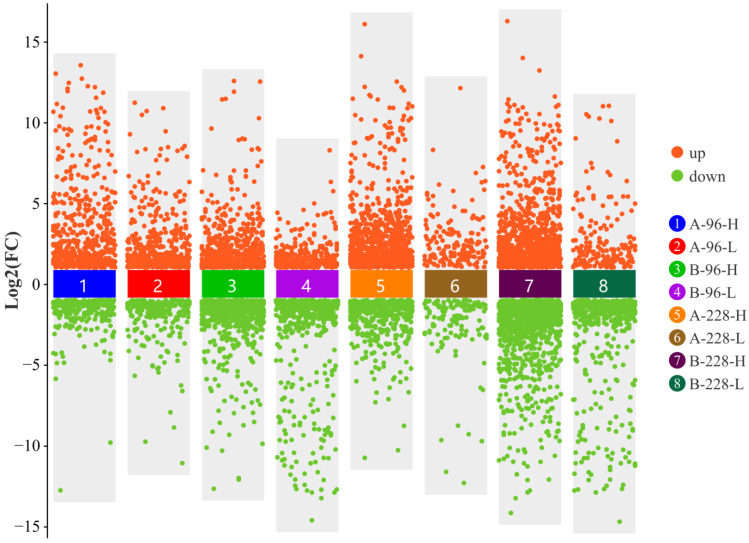
The expression level distribution of DEGs. The maximum expression levels of up-regulated DEGs in high viral load groups surpassed those in low viral load groups.

To identify differentially expressed genes (DEGs), we compared gene expression levels in the A-96-H, A-96-L, A-228-H, and A-228-L groups against the A-0 group, and in the B-96-H, B-96-L, B-228-H, and B-228-L groups against the B-0 group. All of the shrimp in the A-96-H, A-96-L, A-228-H, A-228-L and A-0 groups were from a resistant family. All of the shrimp in the B-96-H, B-96-L, B-228-H, B-228-L and B-0 groups were from a susceptible family. It was found that the quantity of DEGs was greater in the susceptible family than in the resistant family (Figure 1). Groups with a high viral load showed a higher number of DEGs (Figure 2), especially at 228 h post-infection (hpi) (Figure 2C,D). For instance, in the A-228-H group, 890 DEGs were up-regulated and 442 down-regulated, compared to just 169 up-regulated and 128 down-regulated DEGs in the A-228-L group. Similarly, the B-228-H group had 1080 up-regulated and 1414 down-regulated DEGs, surpassing the B-228-L group’s 178 up-regulated and 459 down-regulated DEGs. A further analysis of the DEG expression levels showed that, within the same family and infection period, the maximum expression levels of up-regulated DEGs in high viral load groups surpassed those in low viral load groups. This specific pattern was not mirrored in down-regulated DEGs (Figure 3).

**Figure 4:**
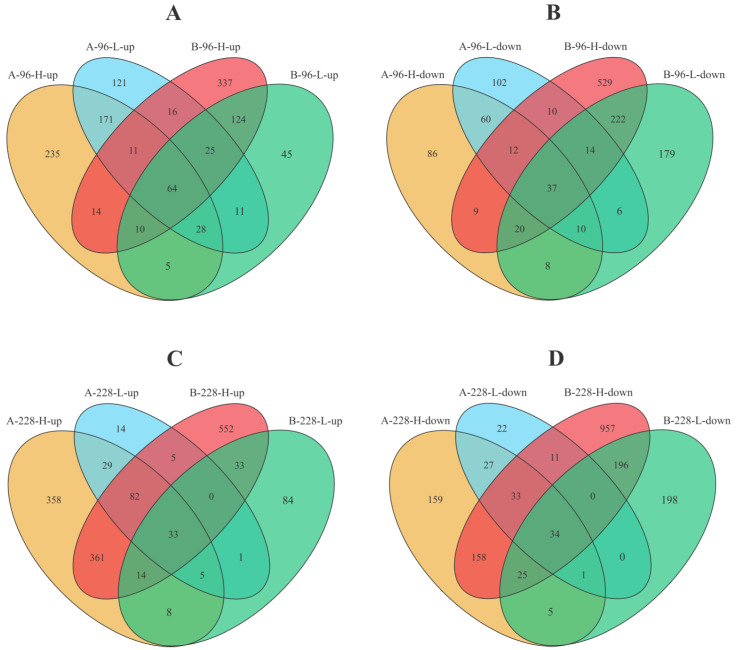
Venn diagram of DEGs across different groups. (**A**) Up-regulated DEGs in groups of A-96-H, A-96-L, B-96-H and B-96-L. Supplementary Table S2 details the 64 DEGs (center of the Venn diagram) present in all contrasts for each group. (**B**) Down-regulated DEGs in groups of A-96-H, A-96-L, B-96-H and B-96-L. Supplementary Table S3 details the 37 DEGs (center of the Venn diagram) present in all contrasts for each group. (**C**) Up-regulated DEGs in groups of A-228-H, A-228-L, B-228-H and B-228-L. Supplementary Table S4 details the 33 DEGs (center of the Venn diagram) present in all contrasts for each group. (**D**) Down-regulated DEGs in groups of A-228-H, A-228-L, B-228-H and B-228-L. Supplementary Table S5 details the 34 DEGs (center of the Venn diagram) present in all contrasts for each group.

Subsequently, to identify underlying universal biological patterns, we went further to identify common DEGs across different resistance families within both high and low viral load groups at two distinct time points (Figure 4). We found that 274 up-regulated DEGs and 119 down-regulated DEGs were repeatedly represented across the A-96-H and A-96-L groups; 223 up-regulated DEGs and 293 down-regulated DEGs were repeatedly represented across the B-96-H and B-96-L groups (Figure 4A,B); 149 up-regulated DEGs and 95 down-regulated DEGs were repeatedly represented across the A-228-H and A-228-L groups; 80 up-regulated DEGs and 255 down-regulated DEGs were repeatedly represented across the B-228-H and B-228-L groups (Figure 4C,D).

In particular, 64 up-regulated DEGs and 37 down-regulated DEGs were repeatedly represented across the A-96-H, A-96-L, B-96-H, and B-96-L groups (Figure 4A,B, and Supplementary Tables S2 and S3). At 228 hpi, 33 up-regulated DEGs and 34 down-regulated DEGs were repeatedly represented across all groups (Figure 4C,D, and Supplementary Tables S4 and S5). These DEGs may play crucial immune functions across families or individuals with varying levels of resistance.

### 2.3. GO and KEGG Pathway Enrichment Analysis

The GO enrichment analysis (Supplementary Figure S1, and Tables S6 and S7) and KEGG enrichment analysis (Supplementary Figure S2, and Tables S8 and S9) of DEGs revealed variations due to the differences in resistance, infection duration, and viral load in *P*. *vannamei*, indicating these factors’ influence on the shrimp transcriptomic profile. Despite this variability, certain consistent patterns emerged. In the GO analysis, the up-regulated DEGs in groups A-96-H, A-96-L, and B-96-L all showed GO:0006457, associated with protein folding, as the most significant term. Meanwhile, GO:0010917, linked to the reduction of the mitochondrial membrane potential, was the most significant for down-regulated DEGs in the A-96-H, A-96-L, A-228-L, and B-228-L groups. Regarding KEGG analysis, ko04141 (Protein processing in endoplasmic reticulum) ranked as the most significant in up-regulated DEGs across the A-96-H, A-96-L, and B-96-L groups. Ko05130 (Pathogenic *Escherichia coli* infection) emerged with the highest significance in both A-228-H and B-228-H groups. Lastly, ko04146 (Peroxisome) was repeatedly the top significant term in down-regulated DEGs within the B-96-H and B-228-H groups.

**Figure 5:**
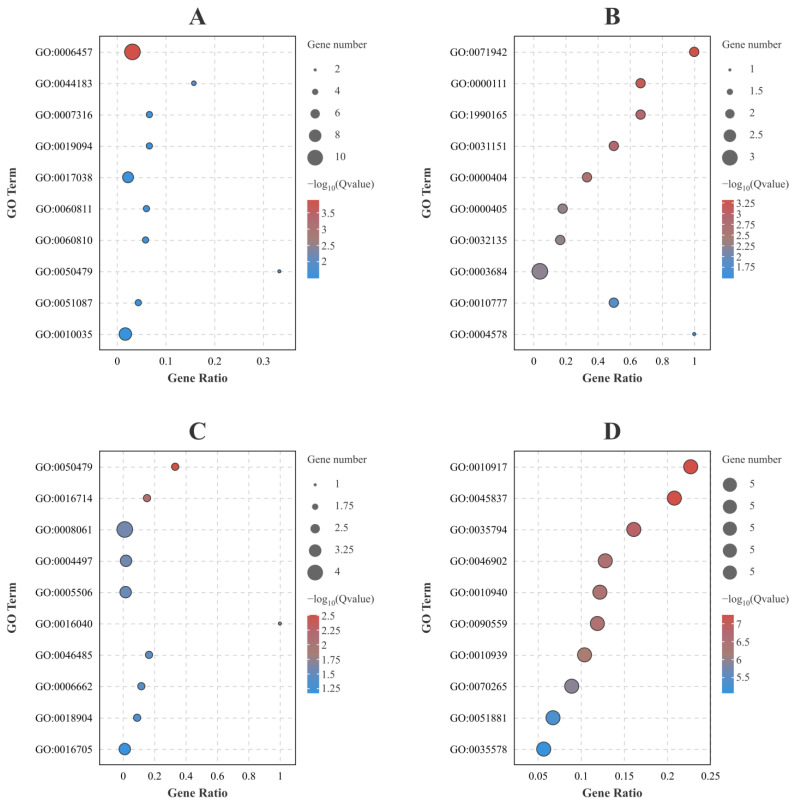
The GO enrichment analysis of DEGs that were repeatedly represented across groups. The size of the dot plot represents the number of genes. (**A**) Up-regulated DEGs repeatedly represented across all groups of A-96-H, A-96-L, B-96-H, and B-96-L. (**B**) Down-regulated DEGs repeatedly represented across all groups of A-96-H, A-96-L, B-96-H, and B-96-L. (**C**) Up-regulated DEGs repeatedly represented across all groups of A-228-H, A-228-L, B-228-H, and B-228-L. (**D**) Down-regulated DEGs repeatedly represented across all groups of A-228-H, A-228-L, B-228-H, and B-228-L.

**Figure 6:**
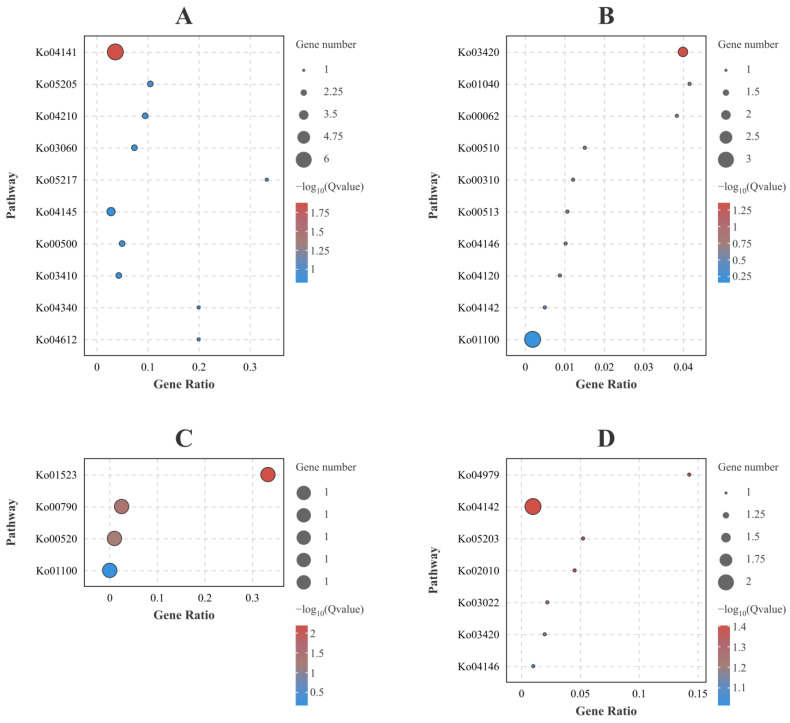
The KEGG enrichment analysis of DEGs that were repeatedly represented across groups. The size of the dot plot represents the number of genes. (**A**) Up-regulated DEGs that were repeatedly represented across all groups of A-96-H, A-96-L, B-96-H, and B-96-L. (**B**) Down-regulated DEGs that were repeatedly represented across all groups of A-96-H, A-96-L, B-96-H, and B-96-L. (**C**) Up-regulated DEGs that were repeatedly represented across all groups of A-228-H, A-228-L, B-228-H, and B-228-L. (**D**) Down-regulated DEGs that were repeatedly represented across all groups of A-228-H, A-228-L, B-228-H, and B-228-L.

The GO and KEGG analyses of common DEGs across different resistance families within both high and low viral load groups are shown in Figure 5 and Figure 6, and Supplementary Tables S10 and S11. At 96 hpi, the top-ranking GO and KEGG terms for the up-regulated DEGs were GO:0006457 (Protein folding) (Figure 5A) and ko04141 (Protein processing in the endoplasmic reticulum) (Figure 6A), respectively. For the down-regulated DEGs, GO:0071942 (XPC complex) (Figure 5B) and ko03420 (Nucleotide excision repair) (Figure 6B) appeared as the foremost significant terms. At 228 hpi, GO:0050479 (Glyceryl-ether monooxygenase activity) (Figure 5C) and ko01523 (Antifolate resistance) (Figure 6C) were the leading terms in the up-regulated DEGs. The top-ranked terms for the down-regulated DEGs were GO:0010917 (Negative regulation of mitochondrial membrane potential) (Figure 5D) and ko00030 (Pentose phosphate pathway) (Figure 6D).

For a detailed analysis of the common DEGs in the top-ranking GO and KEGG terms at 96 hpi: Ten up-regulated DEGs were grouped into GO:0006457 (Protein folding), which were MSTRG.11190 (beta-actin), MSTRG.26451 (heat shock protein), ncbi_113802226 (hsp70-binding protein 1-like), ncbi_113806043 (heat shock protein HSP 90-alpha, transcript variant X1), ncbi_113816031 (heat shock protein 60A-like), ncbi_113818647 (activator of 90 kDa heat shock protein ATPase homolog 1-like), ncbi_113818927 (endoplasmic reticulum chaperone BiP-like), ncbi_113819739 (T-complex protein 1 subunit delta-like), ncbi_113821105 (protein disulfide-isomerase A6 homolog) and ncbi_113828837 (tubulin-specific chaperone A-like, transcript variant X1). Six up-regulated DEGs were grouped into ko04141 (Protein processing in endoplasmic reticulum), which were ncbi_113802226 (hsp70-binding protein 1-like), ncbi_113806043 (heat shock protein HSP 90-alpha, transcript variant X1), ncbi_113809516 (protein transport protein Sec61 subunit alpha-like 1), ncbi_113818927 (endoplasmic reticulum chaperone BiP-like), ncbi_113821105 (protein disulfide-isomerase A6 homolog) and MSTRG.26451 (heat shock protein). Two down-regulated DEGs were grouped into GO:0071942 (XPC complex), which were ncbi_113802762 (DNA repair protein complementing XP-C cells homolog) and ncbi_113818888 (DNA repair protein complementing XP-C cells homolog). Two down-regulated DEGs were grouped into ko03420 (Nucleotide excision repair), which were ncbi_113802762 (DNA repair protein complementing XP-C cells homolog) and ncbi_113818888 (DNA repair protein complementing XP-C cells homolog).

For a detailed analysis of the common DEGs in the top-ranking GO and KEGG terms at 228 hpi: Two up-regulated DEGs were grouped into GO:0050479 (Glyceryl-ether monooxygenase activity), which were ncbi_113816761 (alkylglycerol monooxygenase-like) and ncbi_113825862 (alkylglycerol monooxygenase-like). One up-regulated DEG was grouped into ko01523 (Antifolate resistance), which was MSTRG.23662 (gamma-glutamyl hydrolase-like). Eight down-regulated DEGs were grouped into GO:0010917 (Negative regulation of mitochondrial membrane potential), which were ncbi_113806454 (heme-binding protein 2), ncbi_113810344 (heme-binding protein 2-like), ncbi_113810377 (heme-binding protein 2-like), ncbi_113813866 (heme-binding protein 2-like, transcript variant X1), ncbi_113813867 (heme-binding protein 2-like), ncbi_113813869 (heme-binding protein 2-like), ncbi_113813873 (heme-binding protein 2-like) and ncbi_113813875 (heme-binding protein 2-like). Two down-regulated DEGs were grouped into ko00030 (Pentose phosphate pathway), which were ncbi_113826969 (regucalcin-like) and MSTRG.22444 (regucalcin-like).

### 2.4. Verification Using Real-Time RT-PCR

**Figure 7:**
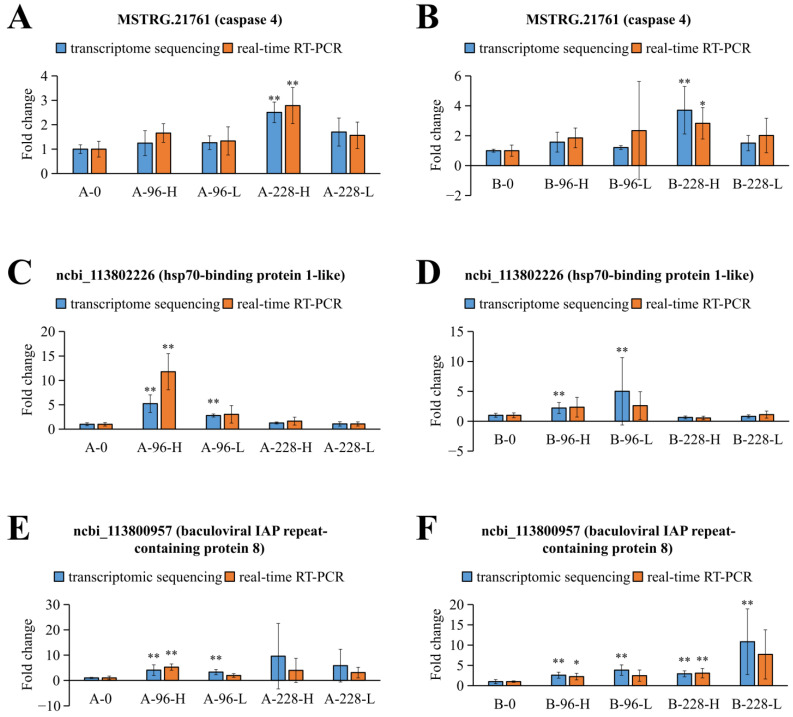
The expression profile of three DEGs in the transcriptome sequencing data, detected using real-time RT-PCR with the same samples. The bars correspond to standard deviation of the means. One asterisk means *p* < 0.05. Two asterisks mean *p* < 0.01. (**A**) MSTRG.21761 (caspase 4) in resistant family. (**B**) MSTRG.21761 (caspase 4) in susceptible family. (**C**) ncbi_113802226 (hsp70-binding protein 1-like) in resistant family. (**D**) ncbi_113802226 (hsp70-binding protein 1-like) in susceptible family. (**E**) ncbi_113800957 (baculoviral IAP repeat-containing protein 8) in resistant family. (**F**) ncbi_113800957 (baculoviral IAP repeat-containing protein 8) in susceptible family.

To corroborate the transcriptome sequencing findings, real-time RT-PCR validation was performed on three randomly chosen DEGs: MSTRG.21761 (caspase 4), ncbi_113802226 (hsp70-binding protein 1-like), and ncbi_113800957 (baculoviral IAP repeat-containing protein 8). The expression levels of these DEGs, as depicted in Figure 7, were largely aligned with the initial transcriptome sequencing results.

**Figure 8:**
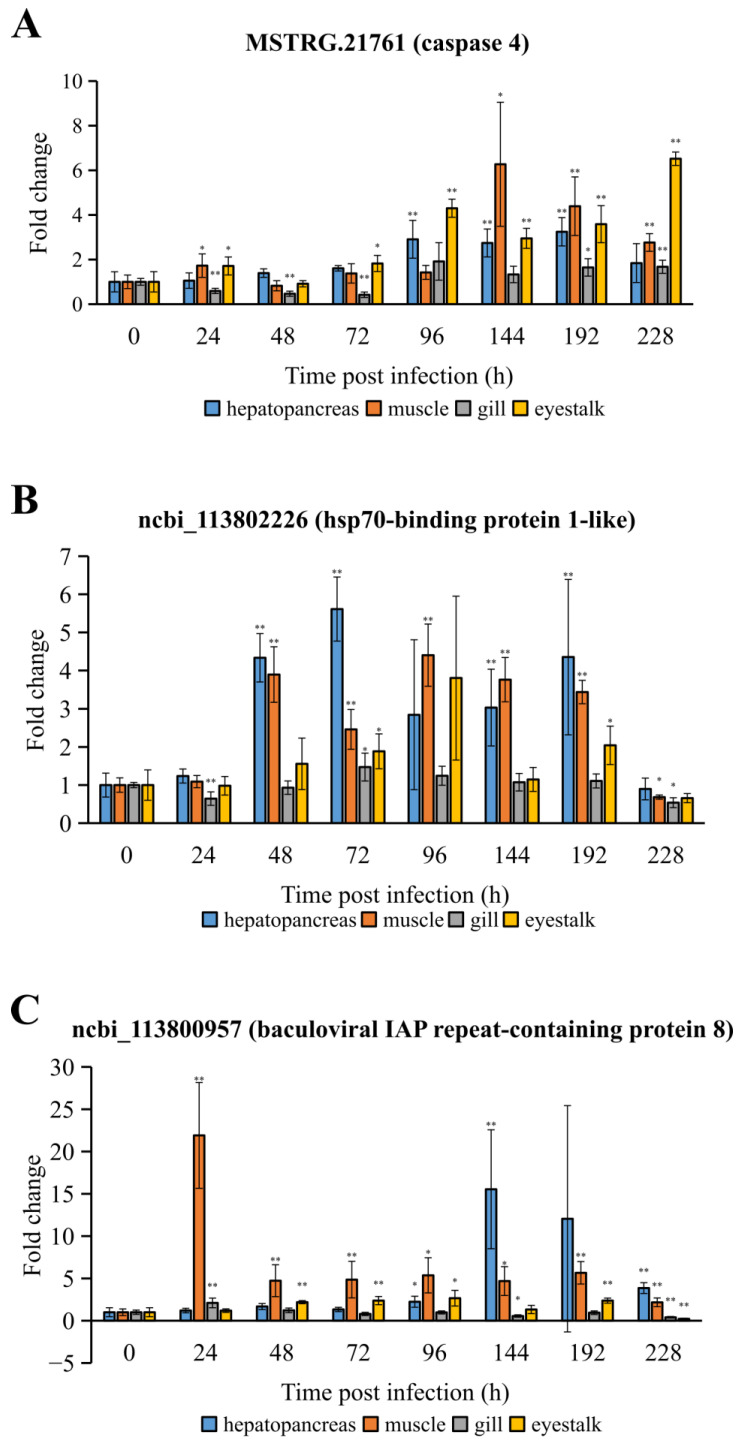
The expression profile of three DEGs further characterized by real-time RT-PCR on samples from a different family than the ones used for transcriptome sequencing. The bars correspond to standard deviation of the means. One asterisk means *p* < 0.05. Two asterisks mean *p* < 0.01. (**A**) MSTRG.21761 (caspase 4). (**B**) ncbi_113802226 (hsp70-binding protein 1-like). (**C**) ncbi_113800957 (baculoviral IAP repeat-containing protein 8).

In the second part of our real-time RT-PCR experiment, we employed shrimp from a family distinct from those in the transcriptome sequencing. The results, displayed in Figure 8, indicated significant changes in the expression levels of all three DEGs post WSSV infection across various tissues, including hepatopancreas, muscle, gill, and eyestalk.

## 3. Discussion

The current study uncovers that *P*. *vannamei* individuals with a higher viral load of WSSV show more DEGs than those with a lower viral load, even when exposed to the virus for the same time. This suggests that not only the duration of infection, but the extent of WSSV replication as well, primarily drives the shrimp transcriptomic response. Additionally, a comparative analysis of gene expression profiles between susceptible and resistant shrimp families uncovered that the susceptible family exhibits a higher frequency of DEGs than the resistant family. It indicates that the different resistant ability of the family line also contributes to the observed differences at the transcriptome level in *P*. *vannamei* infected with WSSV.

Common DEGs found across samples with different viral loads and from various family lines are vital and warrant in-depth study. At 96 hpi, numerous up-regulated DEGs were found to correspond to molecular chaperones, such as heat shock protein 60A, HSP70-binding protein, heat shock protein 90, activator of 90 kDa heat shock protein ATPase homolog 1, endoplasmic reticulum chaperone BiP, and tubulin-specific chaperone A. The results suggested that molecular chaperones not only assist in protein homeostasis but may also play specific roles in the immune response against WSSV. The research highlighted that the heat shock protein 60 expression in *P*. *vannamei* was influenced by WSSV and *Vibrio alginolyticus* infections [9]. Moreover, higher expressions of heat shock proteins 60, 70, and 90 have been documented in *Macrobrachium rosenbergii* infection with WSSV, *Aeromonas hydrophilla* and *Vibrio harveyi* [10]. Hyperthermic treatments increased heat shock protein 70 levels, subsequently diminishing gill-associated virus replication [11]. Heat shock protein 60 could facilitate hepatitis B virus X protein-induced apoptosis [12]. Additionally, heat shock protein 60 could enhance the activity of p38 MAP kinase and regulate bacterial immunity [13]. Furthermore, there are several DEGs involved in the cytoskeletal and associated proteins, such as beta-actin, tubulin–tyrosine ligase-like protein 12, transcript variant X1, tubulin alpha-3 chain, and tubulin-specific chaperone A-like, transcript variant X1. It was reported that beta-actin was involved in the replication process of the classical swine fever virus, and its knockdown reduced the viral RNA copies and titers [14]. In hepatocellular carcinoma tissues infected with hepatitis C virus, a significantly up-regulated 42 kDa tubulin alpha-6 chain fragment was detected [15]. This suggests that cytoskeletal and associated proteins may be related to the replication process of WSSV, and are worthy of further study.

Many common up-regulated DEGs at 228 hpi are associated with pattern recognition receptors, such as C-type lectin domain family 4 member F-like and C-type mannose receptor 2-like and ladderlectin-like, which are all C-type lectins. Numerous studies highlight the role of C-type lectins in immunity in crustaceans. For instance, MjLecA, MjLecB, and MjLecC from *Marsupenaeus japonicus* bind to viral envelope proteins, inhibiting WSSV from infecting hemocytes [16]. Similarly, PcLec-1 and PcLec-2 from *Procambarus clarkii* were involved in combatting bacterial and viral infections [17,18]. Additionally, MjGCTL from *M*. *japonicus* demonstrated bacterial agglutination capabilities [19].

The results indicate an increase in GO and KEGG terms related to protein synthesis and processing among the up-regulated DEGs at 96 hpi, suggesting new protein synthesis within the shrimp following WSSV infection. Determining whether this process benefits the shrimp immune defense or aids in viral replication requires further investigation. Concurrently, the enrichment of GO and KEGG terms of down-regulated DEGs at 96 hpi showed an increase in DNA repair terms. Since viral infection can also alter the host’s DNA, it is hypothesized that this phenomenon could be due to the virus interfering with the host’s normal DNA repair mechanisms, thereby creating a cellular environment more conducive to viral replication. However, this hypothesis requires validation through more detailed research. At 228 hpi, the GO terms of up-regulated DEGs suggest an enrichment in oxidoreductase and monooxygenase activities, indicating that WSSV infection likely influences the activation of redox processes. These processes are essential for synthesizing and decomposing organic compounds, energy metabolism, and cellular signaling. Concurrently, the GO analysis of down-regulated DEGs at 228 hpi points to the negative regulation of the mitochondrial membrane potential alongside the positive regulation of the membrane permeability. This pattern implies that WSSV infection might influence ATP synthesis and apoptosis by influencing the mitochondrial membrane potential and membrane permeability. We found that the categories of DEGs differed at various WSSV infection time points, aligning with existing transcriptomic studies that report gene expression changes as dynamic and time-variant [7]. These results highlight the complexity of the interactions between the host and pathogen. Furthermore, a separate study on transcriptomic sequencing after WSSV infection revealed that genes associated with the immune response, apoptosis, cytoskeleton, and antioxidants play a role in reacting to viral infection [8]. This discovery is consistent with the types of DEGs identified in our research, indicating a similarity in the implicated gene categories.

The three DEGs selected for validation were chosen based on two criteria: first, their differential expression was confirmed in the transcriptome sequencing data; second, they are hypothesized to play roles in the host–virus interaction. Figure 7 demonstrates that DEGs exhibit unique expression patterns in resistant versus susceptible shrimp families. The expression level of baculoviral IAP repeat-containing protein 8 significantly increases in the B-228-L group but not in the A-228-L group. This indicates that the expression pattern of this gene is not strictly similar across shrimp families with different resistance levels. Furthermore, the expression level at 228 hpi in the susceptible family suggests a negative correlation with WSSV replication levels at this time point. However, it is vital to acknowledge the limited data available in this study. Whether these results represent a general rule requires further verification through more extensive experimentation in the future. Additionally, the distinct expression characteristic of the hsp70-binding protein 1-like gene suggests a significant role in the hepatopancreas at 96 h post WSSV infection, possibly losing its function by 228 hpi. In WSSV-infected *P*. *vannamei* from a family distinct from those in the transcriptome sequencing, the significant up-regulation of the three DEGs confirmed by a real-time RT-PCR further validates the accuracy of the transcriptome sequencing performed in this experiment. Additionally, existing research reports have also demonstrated that these three DEGs may possess important immune functions, making them worthy of further investigation. It was reported that caspase 4 could regulate pyroptosis and interleukin-1β synthesis in human macrophages during dengue virus serotype-2 infection [20]. Similarly, it was found that caspase was up-regulated in the hepatopancreas of WSSV-infected *M*. *japonicus*, and was associated with WSSV replication [21]. Moreover, HSP70-binding protein 1 has been shown to hinder HIV-1 replication by blocking the NF-κB-mediated viral transcription activation [22]. Additionally, bacterial and viral infections in *Ictalurus punctatus* could alter the expression level of baculoviral IAP repeat-containing 7, suggesting that this gene may have immune function [23]. It is noteworthy that baculoviral IAP repeat-containing protein 8 exhibited the highest levels of up-regulation in muscle and hepatopancreatic tissues following WSSV infection, reaching more than 20-fold in muscle at 24 hpi and over 15-fold in hepatopancreas at 144 hpi compared to controls. We speculate that baculoviral IAP repeat-containing protein 8 may play a role in inhibiting apoptosis during the WSSV infection process, which may benefit either the shrimp or the WSSV. This hypothesis requires further experimental validation. Moreover, we have also observed that the expression levels of caspase 4 are higher between 96 and 228 h following WSSV infection, leading us to speculate that this gene plays a more crucial role in the later stages of WSSV infection. The expression profile of the hsp70-binding protein 1-like gene suggests that its expression levels in the hepatopancreas and muscle tissues are more substantially influenced by WSSV infection.

In conclusion, we found that, even when exposed to WSSV for the same duration, the extent of WSSV replication greatly drives the shrimp transcriptomic response. The varying levels of resistance across different family lines also contribute to the observed transcriptome differences. Notably, there were common DEGs identified across samples with varying viral load and from different family lines, highlighting the complexity of the response and underlining the need for further research.

## 4. Materials and Methods

### 4.1. Animals

The shrimp specimens utilized in the study were all cultured under identical conditions within the same facility, and were from a population of artificially selected *P*. *vannamei* bred by the Chinese Academy of Fishery Sciences, Yellow Sea Fisheries Research Institute. The culture condition involved maintaining a salinity of 30 parts per thousand and a temperature of 28 °C in the shrimp aquaculture water. In order to maintain water quality, the shrimp were fed regularly with commercial feed purchased from the Charoen Pokphand Group, which includes 42% crude protein. Moreover, we closely monitored water quality parameters using a water quality analyzer (model 5B-3B (V11), Beijing Lianhua YongXing Science and Technology Development Co., Ltd., Beijing, China), such as ammonia nitrogen and nitrite, ensuring that ammonia nitrogen was maintained below 0.2 mg/L, and nitrite below 0.2 mg/L. Additionally, we measured the pH with a pH meter (model PH828, Dongguan Wanchuang Electronic Products Co., Ltd., Dongguan, China), maintaining it around 8.2 to ensure optimal conditions for the shrimp. Before commencing the experiment, the shrimp population earmarked for use underwent thorough testing to ensure the absence of common pathogens, such as white spot syndrome virus (WSSV), Enterocytozoon hepatopenaei (EHP), infectious hypodermal and hematopoietic necrosis virus (IHHNV), *Vibrio* causing acute hepatopancreatic necrosis disease (*V*_AHPND_), covert mortality nodavirus (CMNV), infectious myonecrosis virus (IMNV), and decapod iridescent virus 1 (DIV1).

### 4.2. Challenge Test and Sample Collection for Transcriptome Sequencing

For the infection of *P*. *vannamei*, WSSV bait was prepared from minced muscle of WSSV-infected shrimp, enhanced with red food coloring for visibility. The bait’s WSSV load was quantified using TaqMan real-time PCR [24] with an ABI 7500 system (Applied Biosystems, Foster City, CA, USA) and Premix ExTaq™ (Probe qPCR) kits (TaKaRa, Dalian, China). Primer and probe details are provided in Table 1.

We exposed 59 selectively bred *P*. *vannamei* families (30 shrimp each) to the bait during feeding, at a dose of 1 × 10^7^ WSSV particles per shrimp. The families were then divided into three even groups and housed in separate tanks for resistance evaluation based on survival rate analysis.

Based on the results of the aforementioned challenge test, one family (postlarvae age: 101 days) displaying high resistance to WSSV (with a 73% survival rate 5 days post WSSV infection) and another family (postlarvae age: 100 days) exhibiting low resistance to WSSV (with a 23% survival rate 5 days post WSSV infection) were selected as the resistant family and susceptible family, respectively. Each family comprising 600 individuals underwent a re-challenge with WSSV bait containing 1 × 10^7^ WSSV particles. The hepatopancreas and muscle tissues of 5 healthy shrimp individuals, 50 individuals at 96 hpi, and 50 individuals at 228 hpi from each family were collected for transcriptome sequencing sample preparation. Muscle tissue analysis determined the WSSV load in individual shrimp, enabling the selection of specimens with high and low viral loads for hepatopancreas transcriptome sequencing. All sampled shrimp remained alive during testing, with any deceased individuals promptly removed from the aquaculture system.

### 4.3. Viral Load Detection

The WSSV load was determined in the muscle of 50 shrimp at 96 hpi and 228 hpi each, using the TIANamp marine animals DNA kit (Tiangen Biotech Co., Ltd., Beijing, China) for DNA extraction. Subsequent TaqMan real-time PCR quantification [24], performed with an ABI 7500 fluorescence quantitative PCR system (Applied Biosystems, Foster City, CA, USA) and Premix ExTaq™ (Probe qPCR) kit (TaKaRa, Dalian, China), calculated viral load by the WSSV to 18S rRNA gene ratio, facilitating selection of high and low WSSV load shrimp for transcriptome sequencing. Primer and probe details are provided in Table 1.

### 4.4. Transcriptome Sequencing Sample Preparation

Post WSSV load analysis, the top and bottom five individuals in terms of viral load at 96 hpi and 228 hpi from both resistant and susceptible shrimp families were categorized for transcriptome sequencing. Specifically, for the resistant family, individuals at 96 hpi with the highest (1.02 × 10^−2^ ± 9.69 × 10^−4^) and lowest (3.72 × 10^−4^ ± 1.02 × 10^−4^) viral load were sorted into A-96-H and A-96-L groups, whereas those at 228 hpi were sorted into A-228-H (5.12 × 10^−1^ ± 4.90 × 10^−2^) and A-228-L (1.38 × 10^−2^ ± 3.12 × 10^−3^) groups. In the susceptible family, individuals at 96 hpi were categorized into B-96-H (1.40 × 10^0^ ± 3.35 × 10^−1^) and B-96-L (1.10 × 10^−2^ ± 9.68 × 10^−4^) groups, and those at 228 hpi into B-228-H (7.44 × 10^0^ ± 1.24 × 10^0^) and B-228-L (3.48 × 10^−1^ ± 2.87 × 10^−2^) groups. Additionally, ten virus-free shrimp, five from the resistant family and five from the susceptible family, formed the A-0 and B-0 control groups.

The shrimp were quickly euthanized in liquid nitrogen, and hepatopancreas tissue from each group (A-0, A-96-H, A-96-L, A-228-H, A-228-L, B-0, B-96-H, B-96-L, B-228-H, and B-228-L) which was harvested from the selected individuals, yielded samples for RNA extraction and subsequent transcriptome sequencing. For each group, we included 5 biological replicates, leading to a total of 50 samples being sequenced independently.

### 4.5. RNA Extraction, Library Construction and Sequencing

Transcriptome sequencing was conducted on hepatopancreas samples from 50 individuals across ten groups: A-0, A-96-H, A-96-L, A-228-H, A-228-L, B-0, B-96-H, B-96-L, B-228-H, and B-228-L. We extracted total RNA using TRIzol (Invitrogen, Carlsbad, CA, USA) and assessed RNA quality and concentration with RNase-free agarose gel electrophoresis and the Agilent 2100 Bioanalyzer (Agilent Technologies, Palo Alto, CA, USA). Following mRNA enrichment with Oligo(dT) beads and fragmentation, we synthesized cDNA using the NEBNext Ultra RNA Library Prep Kit (New England Biolabs, Ipswich, MA, USA). This cDNA underwent end-repair, A-tailing, and adapter ligation before purification with AMPure XP Beads and size selection via agarose gel. After PCR amplification, the resulting cDNA library was sequenced to generate 150 bp paired-end sequences on Illumina Novaseq 6000 by Gene Denovo Biotechnology Co. (Guangzhou, China).

### 4.6. Data Analysis

To generate high-quality clean reads, we filtered the sequencing data using fastp (version 0.18.0) [25]. This included removing adapter sequences, discarding reads with over 10% Ns, and eliminating reads where over 50% of bases had a Q-value ≤ 20. Quality of the resulting clean data was evaluated based on Q20, Q30, and GC content. We mapped the paired-end clean reads to the *P*. *vannamei* reference genome (NCBI: ASM378908v1) utilizing HISAT2 (version 2.1.0) [26]. The mapping was performed with the parameter “-rna-strandness RF” and all other parameters were retained at their default settings.

### 4.7. DEGs Analysis

Gene expression levels were quantified as fragments per kilobase of transcript per million mapped reads (FPKM). We utilized DESeq2 (version 1.20.0) software [27] to identify DEGs by comparing the WSSV-infected experimental groups (A-96-H, A-96-L, A-228-H, A-228-L, B-96-H, B-96-L, B-228-H, B-228-L) with their respective control groups (A-0 and B-0). In our analysis, we classified genes as significantly differentially expressed if they had a false discovery rate (FDR) below 0.05 and an absolute fold change of at least 2. This was followed by Gene Ontology (GO) (database v3.14.0) and Kyoto Encyclopedia of Genes and Genomes (KEGG) pathway enrichment analysis.

### 4.8. Validation of Transcriptomic DEGs via Real-Time RT-PCR

To confirm the results obtained from the transcriptomic data for DEGs, we performed real-time RT-PCR to analyze the expression profiles of DEGs. The real-time RT-PCR validation experiment included two parts. The first part involved using real-time RT-PCR technology to detect the DEGs expression levels within the same samples used for transcriptome sequencing.

In the second part, we carried out a follow-up WSSV infection experiment on shrimp from a new family, a different lineage from those used in the transcriptome sequencing. These shrimp, with an average length of (6.1 ± 0.4) cm, were exposed to bait containing 1 × 10^7^ WSSV particles. At each time point—24, 48, 72, 96, 144, 192, and 228 hpi—we sampled the hepatopancreas, muscle, gill, and eyestalk from five infected shrimp, respectively. In parallel, tissue samples were also obtained from five uninfected control shrimps to serve as a baseline comparison.

The ABI 7500 fluorescence quantitative PCR system (Applied Biosystems, Foster City, CA, USA) and a SYBR^®^ Premix Ex Taq™ II kit (TaKaRa, Dalian, China) were used in the experiment, with the 18S rRNA serving as an internal control. The primer sequences utilized are presented in Table 1. Three parallel experiments were conducted in the setup, and each PCR reaction mixture (20 μL) was composed of 0.8 μL of each primer (10 mM), 10 μL SYBR^®^ Premix Ex Taq™ II (TaKaRa, Dalian, China), 20 ng cDNA template, and 0.4 μL ROX Reference Dye II (TaKaRa, Dalian, China). The cycling parameters consisted of an initial denaturation at 95 °C for 30 s, followed by 40 cycles of denaturation at 95 °C for 5 s and annealing/extension at 60 °C for 34 s, with a final dissociation step. The 2^−ΔΔCt^ method was used to analyze data, and statistical analysis was carried out using an unpaired two-tailed *t*-test.

## Figures and Tables

**Table 1:** Primers and probes information.

Designation	Sequences (5′-3′)	Usage
WSSV F	TGGTCCCGTCCTCATCTCAG	WSSV load detection
WSSV R	GCTGCCTTGCCGGAAATTA	WSSV load detection
WSSV probe	AGCCATGAAGAATGCCGTCTATCACACA	WSSV load detection
18S rRNA gene F	AGCAGGCTGGTTTTTGCTTA	WSSV load detection
18S rRNA gene R	GTTCCGAAAAACCGACAAAA	WSSV load detection
18S rRNA gene probe	CCCGAATGGTCGTGCATGGA	WSSV load detection
caspase 4 F	GCGGCCAAGGACCTCACTAA	gene expression level detection
caspase 4 R	TCGGCTGTGGGGTCAACTTT	gene expression level detection
hsp70-binding protein 1-like F	ATCCACGACGGCTTCGAAAT	gene expression level detection
hsp70-binding protein 1-like R	TGTGTAGGGCCCGTGGTATC	gene expression level detection
baculoviral IAP repeat-containing protein 8 F	TGGCATCTCCGCACTGTCAT	gene expression level detection
baculoviral IAP repeat-containing protein 8 R	CGGGTCTTCAGTCTCGCCTT	gene expression level detection
18S rRNA F	TATACGCTAGTGGAGCTGGAA	gene expression level detection
18S rRNA R	GGGGAGGTAGTGACGAAAAAT	gene expression level detection

## Data Availability

The data presented in this study are available upon request from the corresponding author.

## Supplementary Materials

The following supporting information can be downloaded at: https://www.mdpi.com/article/10.3390/ijms25094961/s1.
