# Supplementary material for: Comparative Transcriptomic Analysis of WSSV-Challenged Penaeus vannamei with Variable Resistance Levels
Source: Int J Mol Sci. 2024 May 2;25(9):4961. doi: 10.3390/ijms25094961 (PMC11084523; doi:10.3390/ijms25094961)
Supplement: Supplementary file 1 [file ijms-25-04961-s001.zip › Supplementary Figures.pdf]

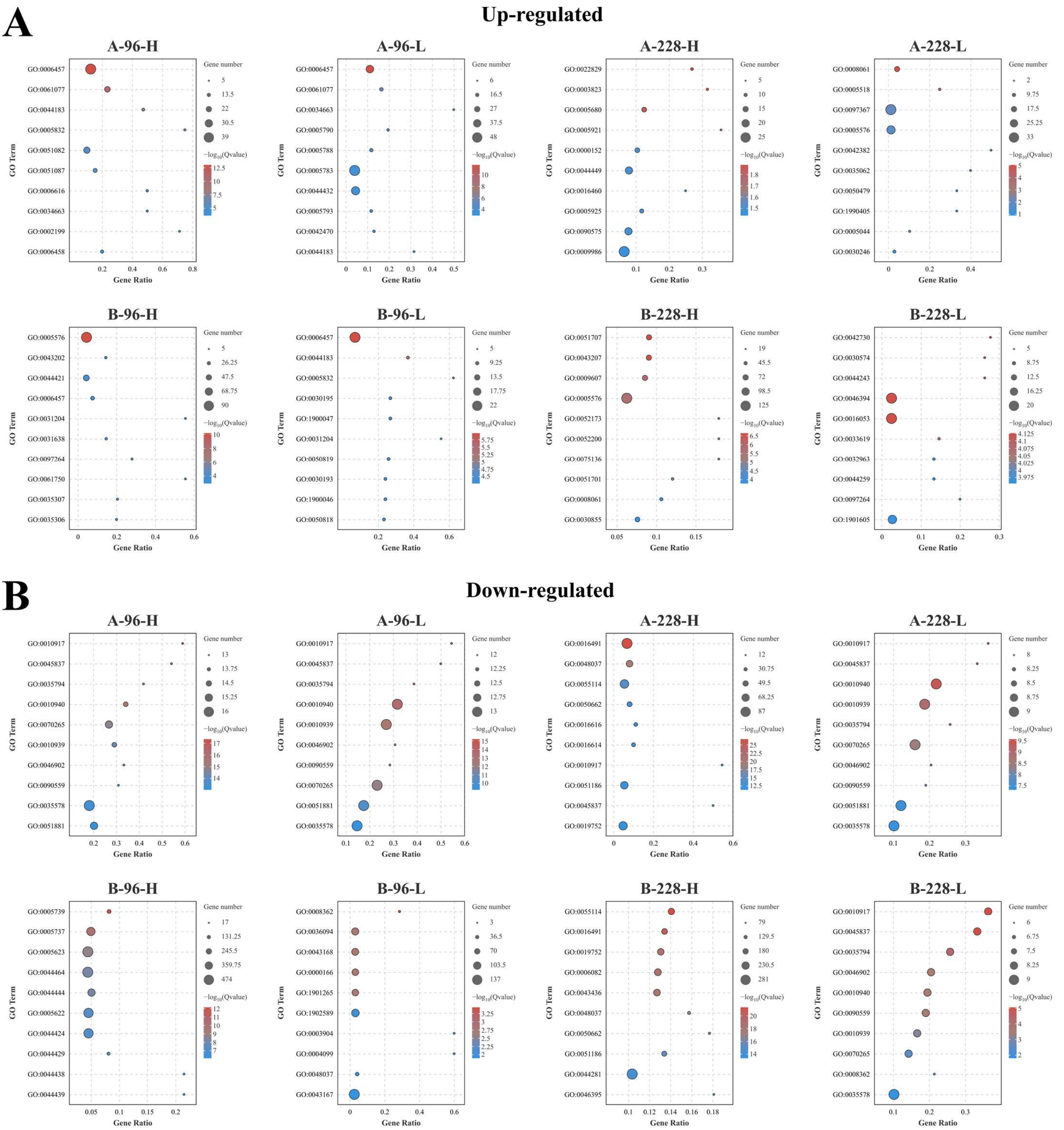

Supplementary Figure S1. The GO enrichment analysis of DEGs. The size of the dot plot represents the number of genes. **(A)** Up-regulated DEGs. **(B)** Down-regulated DEGs.

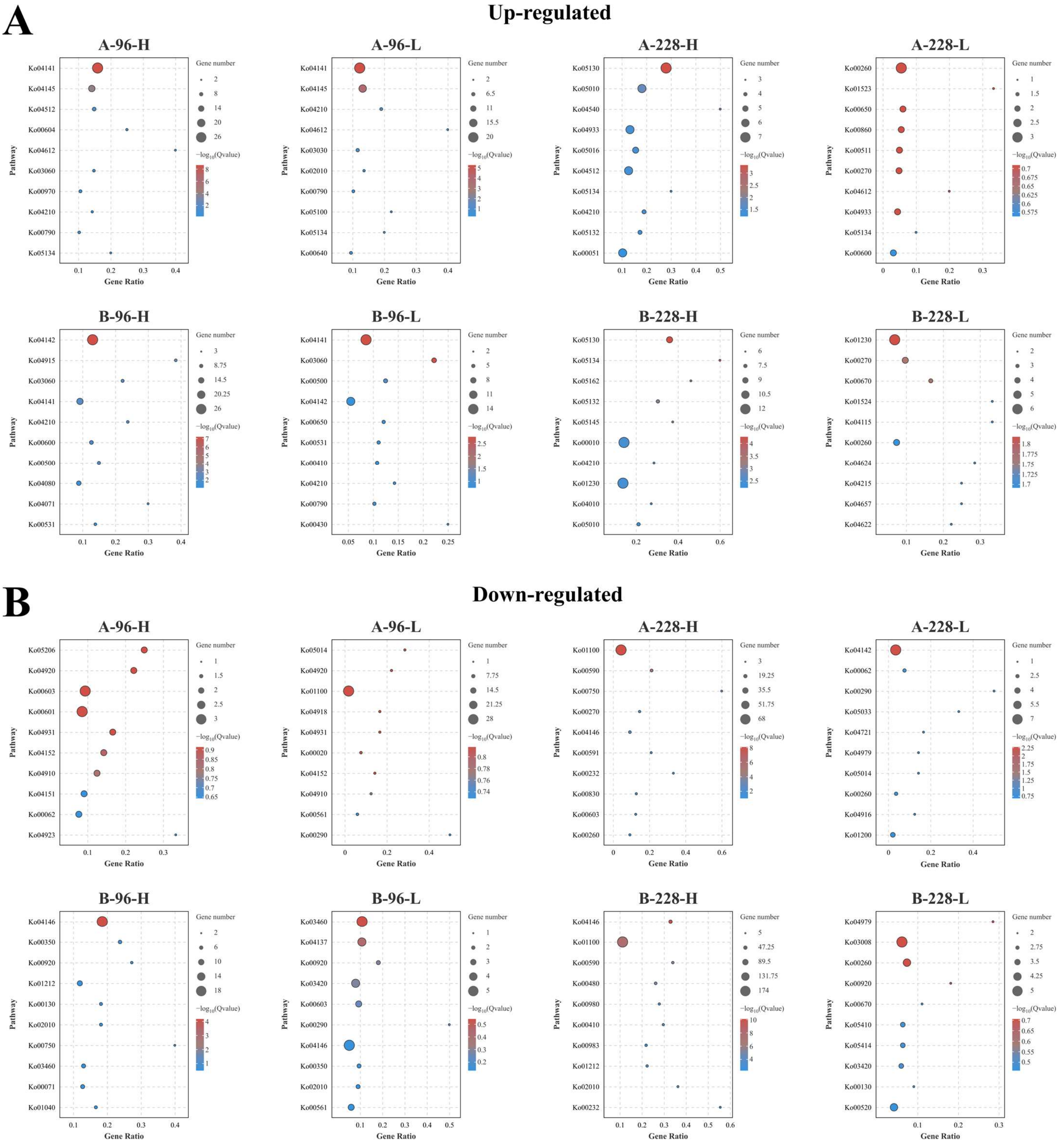

Supplementary Figure S2. The KEGG enrichment analysis of DEGs. The size of the dot plot represents the number of genes. **(A)** Up-regulated DEGs. **(B)** Down-regulated DEGs.
