# Supplementary material for: Comparative Transcriptomic Analysis of WSSV-Challenged Penaeus vannamei with Variable Resistance Levels
Source: Int J Mol Sci. 2024 May 2;25(9):4961. doi: 10.3390/ijms25094961 (PMC11084523; doi:10.3390/ijms25094961)
Supplement: Supplementary file 1 [file ijms-25-04961-s001.zip › Supplementary Tables.pdf]

Supplementary Table S1. Sequencing data statistics.

Group	Sample	Raw reads	Raw bases	Clean reads	Clean bases	Q20(%)	Q30(%)	GC(%)	Mapped(%)
A-0	A-0-1	43844100	6576615000	43567220	6480817532	97.87	94.11	49.52	88.13
	A-0-2	37933546	5690031900	37669964	5597991421	97.93	94.19	49.29	89.57
	A-0-3	43519916	6527987400	43203516	6426788366	98.05	94.54	49.74	89.39
	A-0-4	41864274	6279641100	41583970	6184089817	98.09	94.66	50.15	89.57
	A-0-5	41855012	6278251800	41552530	6194428494	98.05	94.55	50.48	90.10
B-0	B-0-1	42578914	6386837100	42283936	6293252539	98.09	94.60	48.85	90.60
	B-0-2	42817946	6422691900	42533742	6322785859	98.12	94.62	48.42	89.17
	B-0-3	38447676	5767151400	38178990	5673028533	98.03	94.53	49.83	89.51
	B-0-4	40280586	6042087900	40028640	5955359982	98.14	94.65	48.66	90.43
	B-0-5	44968398	6745259700	44670392	6652825504	97.84	93.98	49.30	89.58
A-96-H	A-96-H-1	39565176	5934776400	39305570	5859246008	97.54	93.31	51.62	87.80
	A-96-H-2	39975640	5996346000	39730882	5924105673	98.00	94.41	50.67	88.67
	A-96-H-3	40744800	6111720000	40481200	6019633831	98.06	94.51	49.23	89.22
	A-96-H-4	37871976	5680796400	37598066	5592665814	98.09	94.66	49.55	88.76
	A-96-H-5	41067314	6160097100	40786036	6082283100	98.08	94.60	49.35	88.98
A-96-L	A-96-L-1	41370544	6205581600	41069072	6105701529	97.49	93.35	50.11	87.83
	A-96-L-2	39071592	5860738800	38809570	5791061837	97.86	94.11	50.74	88.67
	A-96-L-3	38367876	5755181400	38128590	5675669624	98.17	94.79	49.70	88.89
	A-96-L-4	38391096	5758664400	38145596	5673143054	97.85	94.06	51.30	88.01
	A-96-L-5	42665708	6399856200	42340586	6308800260	97.49	93.37	49.79	88.06
B-96-H	B-96-H-1	39468814	5920322100	39208520	5827470413	98.01	94.35	52.04	87.51
	B-96-H-2	42911516	6436727400	42614184	6350671588	97.78	93.97	51.84	88.23
	B-96-H-3	46668376	7000256400	46398788	6906155794	98.26	95.02	52.25	89.66
	B-96-H-4	40850500	6127575000	40534394	6013581610	97.84	94.02	51.90	87.84
	B-96-H-5	40393630	6059044500	40162534	5978810903	98.12	94.72	48.77	88.49
B-96-L	B-96-L-1	39954706	5993205900	39713046	5893276112	98.18	94.76	49.47	88.81
	B-96-L-2	39934202	5990130300	39658798	5899388860	98.07	94.49	49.31	87.50
	B-96-L-3	41489624	6223443600	41217054	6120990723	98.14	94.70	51.05	88.10
	B-96-L-4	40853372	6128005800	40583284	6031959608	97.87	94.19	52.72	88.55
	B-96-L-5	41543078	6231461700	41263240	6124544246	98.13	94.69	52.66	88.61
A-228-H	A-228-H-1	37054554	5558183100	36797424	5472368024	97.92	94.23	50.71	88.16
	A-228-H-2	43282978	6492446700	42985804	6413144678	97.98	94.34	49.97	88.60
	A-228-H-3	42940524	6441078600	42651258	6350883562	98.05	94.54	49.85	88.54
	A-228-H-4	40933316	6139997400	40656968	6055356701	98.10	94.73	50.25	88.48
	A-228-H-5	41501864	6225279600	41226078	6133952023	97.92	94.19	50.35	88.80
A-228-L	A-228-L-1	36726756	5509013400	36504206	5425912126	98.14	94.65	49.65	89.63
	A-228-L-2	38425550	5763832500	38154156	5679593439	97.75	93.81	49.66	88.23
	A-228-L-3	45265998	6789899700	44942612	6673925554	97.51	93.25	50.41	88.04
	A-228-L-4	41008050	6151207500	40720812	6040398891	97.92	94.26	51.26	88.56
	A-228-L-5	37566842	5635026300	37321874	5541799208	98.09	94.61	49.93	88.37
B-228-H	B-228-H-1	38738474	5810771100	38427810	5714336482	97.74	93.89	49.93	88.21
	B-228-H-2	39188982	5878347300	38948306	5794208390	97.86	94.11	49.63	90.83
	B-228-H-3	41996394	6299459100	41711018	6195861796	97.56	93.47	49.73	89.98
	B-228-H-4	45648616	6847292400	45356696	6751954544	98.05	94.51	49.73	91.08
	B-228-H-5	41171058	6175658700	40918938	6083736708	98.22	94.91	49.44	90.36
B-228-L	B-228-L-1	37049536	5557430400	36828298	5480118903	98.01	94.33	49.05	90.46
	B-228-L-2	40478782	6071817300	40225314	5982695779	98.09	94.58	48.23	90.70

B-228-L-3	36361906	5454285900	36126966	5376053148	98.06	94.42	49.00	90.92
B-228-L-4	39318404	5897760600	39069108	5810656078	98.24	94.87	49.75	91.16
B-228-L-5	39784154	5967623100	39520422	5877162714	98.10	94.64	51.14	87.36

Supplementary Table S2. Up-regulated DEGs repeatedly represented across A-96-H, A-96-L, B-96-H and B-96-L groups.

ID	Description
MSTRG.11190	beta-actin
MSTRG.11761	poly [ADP-ribose] polymerase 3-like
MSTRG.11887	Enterin neuropeptide, partial
MSTRG.1433	alkylglycerol monooxygenase-like
MSTRG.15662	-
MSTRG.20145	hedgehog protein
MSTRG.21499	myelin expression factor 2-like isoform X1
MSTRG.26451	heat shock protein
MSTRG.6971	apolipophorins-like
ncbi_113799962	putative glutamate synthase
ncbi_113800159	tubulin--tyrosine ligase-like protein 12, transcript variant X1
ncbi_113800564	D-3-phosphoglycerate dehydrogenase-like
ncbi_113800957	baculoviral IAP repeat-containing protein 8-like
ncbi_113801855	-
ncbi_113802226	hsp70-binding protein 1-like
ncbi_113802729	tyrosine--tRNA ligase, cytoplasmic-like, transcript variant X1
ncbi_113804713	probable E3 ubiquitin-protein ligase DTX2, transcript variant X1
ncbi_113805453	-
ncbi_113805856	-
ncbi_113806043	heat shock protein HSP 90-alpha, transcript variant X1
ncbi_113806190	
ncbi_113807145	alpha,alpha-trehalose-phosphate synthase [UDP-forming]-like
ncbi_113807440	-
ncbi_113807877	-
ncbi_113807878	-
ncbi_113808177	-
ncbi_113808340	pyrimidodiazepine synthase-like
ncbi_113809516	protein transport protein Sec61 subunit alpha-like 1
ncbi_113811176	proton-coupled folate transporter-like
ncbi_113813899	sodium-dependent phosphate transporter 2-like, transcript variant X1
ncbi_113815713	-
ncbi_113815919	glutathione hydrolase 1 proenzyme-like, transcript variant X1
ncbi_113816031	heat shock protein 60A-like
ncbi_113816551	receptor-mediated endocytosis protein 6 homolog, transcript variant X1
ncbi_113816761	alkylglycerol monooxygenase-like
ncbi_113817008	uncharacterized LOC113817008
ncbi_113818334	facilitated trehalose transporter Tret1-like
ncbi_113818647	activator of 90 kDa heat shock protein ATPase homolog 1-like
ncbi_113818694	histone deacetylase 4-like
ncbi_113818779	serine/arginine-rich splicing factor 3-like
ncbi_113818927	endoplasmic reticulum chaperone BiP-like
ncbi_113819739	T-complex protein 1 subunit delta-like
ncbi_113819995	heterogeneous nuclear ribonucleoprotein H3-like
ncbi_113820201	-
ncbi_113820306	-
ncbi_113820689	protein charybde-like
ncbi_113820751	poly [ADP-ribose] polymerase 3-like
ncbi_113821011	multidrug resistance protein 1-like
ncbi_113821105	protein disulfide-isomerase A6 homolog
ncbi_113821188	peptide methionine sulfoxide reductase MsrB-like
ncbi_113822283	protein timeless-like, transcript variant X1
ncbi_113822287	luc7-like protein 3, transcript variant X1
ncbi_113822399	-
ncbi_113822400	sodium- and chloride-dependent GABA transporter ine-like, transcript variant X1
ncbi_113822784	-

ncbi_113824088	glycogenin-1-like
ncbi_113825716	eukaryotic translation initiation factor 4 gamma 1-like, transcript variant X1
ncbi_113825862	alkylglycerol monooxygenase-like
ncbi_113826055	myelin expression factor 2-like
ncbi_113827788	tubulin alpha-3 chain
ncbi_113828369	DNA-binding protein K10-like
ncbi_113828821	programmed cell death protein 7-like
ncbi_113828837	tubulin-specific chaperone A-like, transcript variant X1
ncbi_113830540	serine/arginine repetitive matrix protein 1-like

Supplementary Table S3. Down-regulated DEGs repeatedly represented across A-96-H, A-96-L, B-96-H and B-96-L groups.

ID	Description
MSTRG.10319	TLC domain-containing protein 2-like [*Penaeus vannamei*]
MSTRG.16374	nuclear apoptosis-inducing factor 1-like [*Penaeus monodon*]
MSTRG.18095	-
MSTRG.18808	RNA-binding protein 25-like, partial [*Oncorhynchus gorbuscha*]
ncbi_113802762	DNA repair protein complementing XP-C cells homolog
ncbi_113803380	-
ncbi_113804027	-
ncbi_113804952	ubiquitin carboxyl-terminal hydrolase Usp2-like, transcript variant X1
ncbi_113805022	peroxisomal membrane protein 11A-like, transcript variant X1
ncbi_113805926	ribonuclease Y-like, transcript variant X1
ncbi_113806381	cryptochrome DASH-like, transcript variant X1
ncbi_113806622	HIG1 domain family member 1C-like, transcript variant X1
ncbi_113808549	RING finger protein 37-like, transcript variant X1
ncbi_113813869	heme-binding protein 2-like
ncbi_113814209	TLC domain-containing protein 2-like
ncbi_113816458	protein fem-1 homolog C-like, transcript variant X1
ncbi_113816681	autophagy-related protein 23-like, transcript variant X1
ncbi_113817127	endochitinase A-like
ncbi_113817491	integral membrane protein GPR155-like, transcript variant X1
ncbi_113817807	leucine-rich repeat extensin-like protein 3
ncbi_113817809	histone-lysine N-methyltransferase, H3 lysine-79 specific-like, transcript variant X1
ncbi_113818101	succinate dehydrogenase assembly factor 2, mitochondrial-like
ncbi_113818731	-
ncbi_113818888	DNA repair protein complementing XP-C cells homolog
ncbi_113820493	pyruvate dehydrogenase (acetyl-transferring) kinase, mitochondrial-like, transcript variant X1
ncbi_113820707	protein patched homolog 2-like
ncbi_113820919	chitobiosyldiphosphodolichol beta-mannosyltransferase-like, transcript variant X1
ncbi_113821862	leishmanolysin-like peptidase
ncbi_113822192	sodium-coupled monocarboxylate transporter 1-like
ncbi_113823528	metalloreductase STEAP4-like
ncbi_113824174	facilitated trehalose transporter Tret1-2 homolog, transcript variant X1
ncbi_113824444	probable GPI-anchored adhesin-like protein PGA55, transcript variant X1
ncbi_113824581	kelch-like protein 8
ncbi_113826256	-
ncbi_113826690	acyl-coenzyme A thioesterase 1-like
ncbi_113827721	C-factor-like
ncbi_113830489	lysoplasmalogenase-like protein TMEM86A

Supplementary Table S4. Up-regulated DEGs repeatedly represented across A-228-H, A-228-L, B-228-H and B-228-L groups.

ID	Description
MSTRG.1433	alkylglycerol monooxygenase-like [*Penaeus japonicus*]
MSTRG.18698	atherin-like [*Penaeus vannamei*]
MSTRG.18705	CHK1 checkpoint-like protein, partial [*Helicoverpa armigera*]
MSTRG.23662	gamma-glutamyl hydrolase-like [*Penaeus monodon*]
MSTRG.5817	-
ncbi_113799962	putative glutamate synthase [NADPH]
ncbi_113803696	-

ncbi_113804377	cyclin-G-associated kinase-like
ncbi_113805525	C-type lectin domain family 4 member F-like
ncbi_113805856	-
ncbi_113805857	-
ncbi_113806548	-
ncbi_113806636	-
ncbi_113807570	-
ncbi_113807727	-
ncbi_113808342	C-type mannose receptor 2-like
ncbi_113810052	-
ncbi_113810057	peritrophin-1-like
ncbi_113811552	-
ncbi_113812325	ladderlectin-like
ncbi_113816761	alkylglycerol monooxygenase-like
ncbi_113817247	chitotriosidase-1-like
ncbi_113817597	-
ncbi_113817858	-
ncbi_113820689	protein charybde-like
ncbi_113821705	-
ncbi_113822187	solute carrier family 15 member 2-like, transcript variant X1
ncbi_113822657	-
ncbi_113823073	-
ncbi_113824399	-
ncbi_113824401	-
ncbi_113825862	alkylglycerol monooxygenase-like
ncbi_113826006	-

Supplementary Table S5. Down-regulated DEGs repeatedly represented across A-228-H, A-228-L, B-228-H and B-228-L groups.

ID	Description
MSTRG.18808	RNA-binding protein 25-like, partial [*Oncorhynchus gorbuscha*]
MSTRG.194	protein patched homolog 2-like [*Penaeus vannamei*]
MSTRG.3402	Gag-Pol polyprotein [*Chionoecetes opilio*]
MSTRG.5097	uncharacterized protein LOC113806516 isoform X4 [*Penaeus vannamei*]
MSTRG.8327	-
ncbi_113800718	solute carrier family 43 member 3-like, transcript variant X1
ncbi_113805926	ribonuclease Y-like, transcript variant X1
ncbi_113806381	cryptochrome DASH-like, transcript variant X1
ncbi_113806454	uncharacterized LOC113806454
ncbi_113809949	beta,beta-carotene 15,15′-dioxygenase-like, transcript variant X1
ncbi_113810344	heme-binding protein 2-like
ncbi_113810377	heme-binding protein 2-like
ncbi_113812624	sulfotransferase 1C4-like
ncbi_113813817	apolipoprotein D-like
ncbi_113813869	heme-binding protein 2-like
ncbi_113813873	heme-binding protein 2-like
ncbi_113813874	heme-binding protein 2-like, transcript variant X1
ncbi_113813877	heme-binding protein 2-like
ncbi_113816958	2,4-dienoyl-CoA reductase, mitochondrial-like, transcript variant X1
ncbi_113817491	integral membrane protein GPR155-like, transcript variant X1
ncbi_113818101	succinate dehydrogenase assembly factor 2, mitochondrial-like
ncbi_113818731	uncharacterized LOC113818731
ncbi_113820605	uncharacterized LOC113820605
ncbi_113820707	protein patched homolog 2-like
ncbi_113821862	leishmanolysin-like peptidase
ncbi_113822024	uncharacterized LOC113822024, transcript variant X1
ncbi_113822192	sodium-coupled monocarboxylate transporter 1-like
ncbi_113823528	metalloreductase STEAP4-like
ncbi_113824000	cation diffusion facilitator family protein 1-like, transcript variant X1
ncbi_113824174	facilitated trehalose transporter Tret1-2 homolog, transcript variant X1
ncbi_113825168	ATP-binding cassette sub-family D member 2-like
ncbi_113826256	uncharacterized LOC113826256, transcript variant X1
ncbi_113827299	solute carrier family 22 member 6-A-like

ncbi_113830589	cryptochrome-2-like, transcript variant X1

Supplementary Table S6. The GO enrichment analysis of up-regulated DEGs.

Group	GO ID	Description	Class
A-96-H	GO:0006457	protein folding	Biological Process
	GO:0061077	chaperone-mediated protein folding	Biological Process
	GO:0044183	protein binding involved in protein folding	Molecular Function
	GO:0005832	chaperonin-containing T-complex	Cellular Component
	GO:0051082	unfolded protein binding	Molecular Function
	GO:0051087	chaperone binding	Molecular Function
	GO:0006616	SRP-dependent cotranslational protein targeting to membrane, translocation	Biological Process
	GO:0034663	endoplasmic reticulum chaperone complex	Cellular Component
	GO:0002199	zona pellucida receptor complex	Cellular Component
	GO:0006458	'de novo' protein folding	Biological Process
A-96-L	GO:0006457	protein folding	Biological Process
	GO:0061077	chaperone-mediated protein folding	Biological Process
	GO:0034663	endoplasmic reticulum chaperone complex	Cellular Component
	GO:0005790	smooth endoplasmic reticulum	Cellular Component
	GO:0005788	endoplasmic reticulum lumen	Cellular Component
	GO:0005783	endoplasmic reticulum	Cellular Component
	GO:0044432	endoplasmic reticulum part	Cellular Component
	GO:0005793	endoplasmic reticulum-Golgi intermediate compartment	Cellular Component
	GO:0042470	melanosome	Cellular Component
	GO:0044183	protein binding involved in protein folding	Molecular Function
A-228-H	GO:0022829	wide pore channel activity	Molecular Function
	GO:0003823	antigen binding	Molecular Function
	GO:0005680	anaphase-promoting complex	Cellular Component
	GO:0005921	gap junction	Cellular Component
	GO:0000152	nuclear ubiquitin ligase complex	Cellular Component
	GO:0044449	contractile fiber part	Cellular Component
	GO:0016460	myosin II complex	Cellular Component
	GO:0005925	focal adhesion	Cellular Component
	GO:0090575	RNA polymerase II transcription factor complex	Cellular Component
	GO:0009986	cell surface	Cellular Component
A-228-L	GO:0008061	chitin binding	Molecular Function
	GO:0005518	collagen binding	Molecular Function
	GO:0097367	carbohydrate derivative binding	Molecular Function
	GO:0005576	extracellular region	Cellular Component
	GO:0042382	paraspeckles	Cellular Component
	GO:0035062	omega speckle	Cellular Component
	GO:0050479	glyceryl-ether monooxygenase activity	Molecular Function
	GO:1990405	protein antigen binding	Molecular Function
	GO:0005044	scavenger receptor activity	Molecular Function
	GO:0030246	carbohydrate binding	Molecular Function
B-96-H	GO:0005576	extracellular region	Cellular Component
	GO:0043202	lysosomal lumen	Cellular Component
	GO:0044421	extracellular region part	Cellular Component
	GO:0006457	protein folding	Biological Process
	GO:0031204	posttranslational protein targeting to membrane, translocation	Biological Process

	GO:0031638	zymogen activation	Biological Process
	GO:0097264	self proteolysis	Biological Process
	GO:0061750	acid sphingomyelin phosphodiesterase activity	Biological Process
	GO:0035307	positive regulation of protein dephosphorylation	Biological Process
	GO:0035306	positive regulation of dephosphorylation	Biological Process
B-96-L	GO:0006457	protein folding	Biological Process
	GO:0044183	protein binding involved in protein folding	Molecular Function
	GO:0005832	chaperonin-containing T-complex	Cellular Component
	GO:0030195	negative regulation of blood coagulation	Biological Process
	GO:1900047	negative regulation of hemostasis	Biological Process
	GO:0031204	posttranslational protein targeting to membrane, translocation	Biological Process
	GO:0050819	negative regulation of coagulation	Biological Process
	GO:0030193	regulation of blood coagulation	Biological Process
	GO:1900046	regulation of hemostasis	Biological Process
	GO:0050818	regulation of coagulation	Biological Process
B-228-H	GO:0051707	response to other organism	Biological Process
	GO:0043207	response to external biotic stimulus	Biological Process
	GO:0009607	response to biotic stimulus	Biological Process
	GO:0005576	extracellular region	Cellular Component
	GO:0052173	response to defenses of other organism involved in symbiotic interaction	Biological Process
	GO:0052200	response to host defenses	Biological Process
	GO:0075136	response to host	Biological Process
	GO:0051701	interaction with host	Biological Process
	GO:0008061	chitin binding	Molecular Function
	GO:0030855	epithelial cell differentiation	Biological Process
B-228-L	GO:0042730	fibrinolysis	Biological Process
	GO:0030574	collagen catabolic process	Biological Process
	GO:0044243	multicellular organism catabolic process	Biological Process
	GO:0046394	carboxylic acid biosynthetic process	Biological Process
	GO:0016053	organic acid biosynthetic process	Biological Process
	GO:0033619	membrane protein proteolysis	Biological Process
	GO:0032963	collagen metabolic process	Biological Process
	GO:0044259	multicellular organismal macromolecule metabolic process	Biological Process
	GO:0097264	self proteolysis	Biological Process
	GO:1901605	alpha-amino acid metabolic process	Biological Process

Supplementary Table S7. The GO enrichment analysis of down-regulated DEGs.

Group	GO ID	Description	Class
A-96-H	GO:0010917	negative regulation of mitochondrial membrane potential	Biological Process
	GO:0045837	negative regulation of membrane potential	Biological Process
	GO:0035794	positive regulation of mitochondrial membrane permeability	Biological Process
	GO:0010940	positive regulation of necrotic cell death	Biological Process
	GO:0070265	necrotic cell death	Biological Process
	GO:0010939	regulation of necrotic cell death	Biological Process
	GO:0046902	regulation of mitochondrial membrane permeability	Biological Process
	GO:0090559	regulation of membrane permeability	Biological Process
	GO:0035578	azurophil granule lumen	Cellular Component
	GO:0051881	regulation of mitochondrial membrane potential	Biological Process
A-96-L	GO:0010917	negative regulation of mitochondrial membrane potential	Biological Process

	GO:0045837	negative regulation of membrane potential	Biological Process
	GO:0035794	positive regulation of mitochondrial membrane permeability	Biological Process
	GO:0010940	positive regulation of necrotic cell death	Biological Process
	GO:0010939	regulation of necrotic cell death	Biological Process
	GO:0046902	regulation of mitochondrial membrane permeability	Biological Process
	GO:0090559	regulation of membrane permeability	Biological Process
	GO:0070265	necrotic cell death	Biological Process
	GO:0051881	regulation of mitochondrial membrane potential	Biological Process
	GO:0035578	azurophil granule lumen	Cellular Component
A-228-H	GO:0016491	oxidoreductase activity	Molecular Function
	GO:0048037	cofactor binding	Molecular Function
	GO:0055114	oxidation-reduction process	Biological Process
	GO:0050662	coenzyme binding	Molecular Function
	GO:0016616	oxidoreductase activity, acting on the CH-OH group of donors, NAD or NADP as acceptor	Molecular Function
	GO:0016614	oxidoreductase activity, acting on CH-OH group of donors	Molecular Function
	GO:0010917	negative regulation of mitochondrial membrane potential	Biological Process
	GO:0051186	cofactor metabolic process	Biological Process
	GO:0045837	negative regulation of membrane potential	Biological Process
	GO:0019752	carboxylic acid metabolic process	Biological Process
A-228-L	GO:0010917	negative regulation of mitochondrial membrane potential	Biological Process
	GO:0045837	negative regulation of membrane potential	Biological Process
	GO:0010940	positive regulation of necrotic cell death	Biological Process
	GO:0010939	regulation of necrotic cell death	Biological Process
	GO:0035794	positive regulation of mitochondrial membrane permeability	Biological Process
	GO:0070265	necrotic cell death	Biological Process
	GO:0046902	regulation of mitochondrial membrane permeability	Biological Process
	GO:0090559	regulation of membrane permeability	Biological Process
	GO:0051881	regulation of mitochondrial membrane potential	Biological Process
	GO:0035578	azurophil granule lumen	Cellular Component
B-96-H	GO:0005739	mitochondrion	Cellular Component
	GO:0005737	cytoplasm	Cellular Component
	GO:0005623	cell	Cellular Component
	GO:0044464	cell part	Cellular Component
	GO:0044444	cytoplasmic part	Cellular Component
	GO:0005622	intracellular	Cellular Component
	GO:0044424	intracellular part	Cellular Component
	GO:0044429	mitochondrial part	Cellular Component
	GO:0044438	microbody part	Cellular Component
	GO:0044439	peroxisomal part	Cellular Component
B-96-L	GO:0008362	chitin-based embryonic cuticle biosynthetic process	Biological Process
	GO:0036094	small molecule binding	Molecular Function
	GO:0043168	anion binding	Molecular Function
	GO:0000166	nucleotide binding	Molecular Function
	GO:1901265	nucleoside phosphate binding	Molecular Function
	GO:1902589	single-organism organelle organization	Biological Process
	GO:0003904	deoxyribodipyrimidine photo-lyase activity	Molecular Function
	GO:0004099	chitin deacetylase activity	Molecular Function
	GO:0048037	cofactor binding	Molecular Function

	GO:0043167	ion binding	Molecular Function
B-228-H	GO:0055114	oxidation-reduction process	Biological Process
	GO:0016491	oxidoreductase activity	Molecular Function
	GO:0019752	carboxylic acid metabolic process	Biological Process
	GO:0006082	organic acid metabolic process	Biological Process
	GO:0043436	oxoacid metabolic process	Biological Process
	GO:0048037	cofactor binding	Molecular Function
	GO:0050662	coenzyme binding	Molecular Function
	GO:0051186	cofactor metabolic process	Biological Process
	GO:0044281	small molecule metabolic process	Biological Process
	GO:0046395	carboxylic acid catabolic process	Biological Process
B-228-L	GO:0010917	negative regulation of mitochondrial membrane potential	Biological Process
	GO:0045837	negative regulation of membrane potential	Biological Process
	GO:0035794	positive regulation of mitochondrial membrane permeability	Biological Process
	GO:0046902	regulation of mitochondrial membrane permeability	Biological Process
	GO:0010940	positive regulation of necrotic cell death	Biological Process
	GO:0090559	regulation of membrane permeability	Biological Process
	GO:0010939	regulation of necrotic cell death	Biological Process
	GO:0070265	necrotic cell death	Biological Process
	GO:0008362	chitin-based embryonic cuticle biosynthetic process	Biological Process
	GO:0035578	azurophil granule lumen	Cellular Component

Supplementary Table S8. The KEGG enrichment analysis of up-regulated DEGs.

Group	Pathway ID	Description
A-96-H	ko04141	Protein processing in endoplasmic reticulum
	ko04145	Phagosome
	ko04512	ECM-receptor interaction
	ko00604	Glycosphingolipid biosynthesis - ganglio series
	ko04612	Antigen processing and presentation
	ko03060	Protein export
	ko00970	Aminoacyl-tRNA biosynthesis
	ko04210	Apoptosis
	ko00790	Folate biosynthesis
	ko05134	Legionellosis
A-96-L	ko04141	Protein processing in endoplasmic reticulum
	ko04145	Phagosome
	ko04210	Apoptosis
	ko04612	Antigen processing and presentation
	ko03030	DNA replication
	ko02010	ABC transporters
	ko00790	Folate biosynthesis
	ko05100	Bacterial invasion of epithelial cells
	ko05134	Legionellosis
	ko00640	Propanoate metabolism
A-228-H	ko05130	Pathogenic Escherichia coli infection
	ko05010	Alzheimer disease
	ko04540	Gap junction
	ko04933	AGE-RAGE signaling pathway in diabetic complications
	ko05016	Huntington disease

	ko04512	ECM-receptor interaction
	ko05134	Legionellosis
	ko04210	Apoptosis
	ko05132	Salmonella infection
	ko00051	Fructose and mannose metabolism
A-228-L	ko00260	Glycine, serine and threonine metabolism
	ko01523	Antifolate resistance
	ko00650	Butanoate metabolism
	ko00860	Porphyrin metabolism
	ko00511	Other glycan degradation
	ko00270	Cysteine and methionine metabolism
	ko04612	Antigen processing and presentation
	ko04933	AGE-RAGE signaling pathway in diabetic complications
	ko05134	Legionellosis
	ko00600	Sphingolipid metabolism
B-96-H	ko04142	Lysosome
	ko04915	Estrogen signaling pathway
	ko03060	Protein export
	ko04141	Protein processing in endoplasmic reticulum
	ko04210	Apoptosis
	ko00600	Sphingolipid metabolism
	ko00500	Starch and sucrose metabolism
	ko04080	Neuroactive ligand-receptor interaction
	ko04071	Sphingolipid signaling pathway
	ko00531	Glycosaminoglycan degradation
B-96-L	ko04141	Protein processing in endoplasmic reticulum
	ko03060	Protein export
	ko00500	Starch and sucrose metabolism
	ko04142	Lysosome
	ko00650	Butanoate metabolism
	ko00531	Glycosaminoglycan degradation
	ko00410	beta-Alanine metabolism
	ko04210	Apoptosis
	ko00790	Folate biosynthesis
	ko00430	Taurine and hypotaurine metabolism
B-228-H	ko05130	Pathogenic Escherichia coli infection
	ko05134	Legionellosis
	ko05162	Measles
	ko05132	Salmonella infection
	ko05145	Toxoplasmosis
	ko00010	Glycolysis / Gluconeogenesis
	ko04210	Apoptosis
	ko01230	Biosynthesis of amino acids
	ko04010	MAPK signaling pathway
	ko05010	Alzheimer disease
B-228-L	ko01230	Biosynthesis of amino acids
	ko00270	Cysteine and methionine metabolism
	ko00670	One carbon pool by folate
	ko01524	Platinum drug resistance

	ko04115	p53 signaling pathway
	ko00260	Glycine, serine and threonine metabolism
	ko04624	Toll and Imd signaling pathway
	ko04215	Apoptosis - multiple species
	ko04657	IL-17 signaling pathway
	ko04622	RIG-I-like receptor signaling pathway

Supplementary Table S9. The KEGG enrichment analysis of down-regulated DEGs.

Group	Pathway ID	Description
A-96-H	ko05206	MicroRNAs in cancer
	ko04920	Adipocytokine signaling pathway
	ko00603	Glycosphingolipid biosynthesis - globo and isoglobo series
	ko00601	Glycosphingolipid biosynthesis - lacto and neolacto series
	ko04931	Insulin resistance
	ko04152	AMPK signaling pathway
	ko04910	Insulin signaling pathway
	ko04151	PI3K-Akt signaling pathway
	ko00062	Fatty acid elongation
	ko04923	Regulation of lipolysis in adipocytes
A-96-L	ko05014	Amyotrophic lateral sclerosis
	ko04920	Adipocytokine signaling pathway
	ko01100	Metabolic pathways
	ko04918	Thyroid hormone synthesis
	ko04931	Insulin resistance
	ko00020	Citrate cycle (TCA cycle)
	ko04152	AMPK signaling pathway
	ko04910	Insulin signaling pathway
	ko00561	Glycerolipid metabolism
	ko00290	Valine, leucine and isoleucine biosynthesis
A-228-H	ko01100	Metabolic pathways
	ko00590	Arachidonic acid metabolism
	ko00750	Vitamin B6 metabolism
	ko00270	Cysteine and methionine metabolism
	ko04146	Peroxisome
	ko00591	Linoleic acid metabolism
	ko00232	Caffeine metabolism
	ko00830	Retinol metabolism
	ko00603	Glycosphingolipid biosynthesis - globo and isoglobo series
	ko00260	Glycine, serine and threonine metabolism
A-228-L	ko04142	Lysosome
	ko00062	Fatty acid elongation
	ko00290	Valine, leucine and isoleucine biosynthesis
	ko05033	Nicotine addiction
	ko04721	Synaptic vesicle cycle
	ko04979	Cholesterol metabolism
	ko05014	Amyotrophic lateral sclerosis
	ko00260	Glycine, serine and threonine metabolism
	ko04916	Melanogenesis
	ko01200	Carbon metabolism

B-96-H	ko04146	Peroxisome
	ko00350	Tyrosine metabolism
	ko00920	Sulfur metabolism
	ko01212	Fatty acid metabolism
	ko00130	Ubiquinone and other terpenoid-quinone biosynthesis
	ko02010	ABC transporters
	ko00750	Vitamin B6 metabolism
	ko03460	Fanconi anemia pathway
	ko00071	Fatty acid degradation
	ko01040	Biosynthesis of unsaturated fatty acids
B-96-L	ko03460	Fanconi anemia pathway
	ko04137	Mitophagy - animal
	ko00920	Sulfur metabolism
	ko03420	Nucleotide excision repair
	ko00603	Glycosphingolipid biosynthesis - globo and isoglobo series
	ko00290	Valine, leucine and isoleucine biosynthesis
	ko04146	Peroxisome
	ko00350	Tyrosine metabolism
	ko02010	ABC transporters
	ko00561	Glycerolipid metabolism
B-228-H	ko04146	Peroxisome
	ko01100	Metabolic pathways
	ko00590	Arachidonic acid metabolism
	ko00480	Glutathione metabolism
	ko00980	Metabolism of xenobiotics by cytochrome P450
	ko00410	beta-Alanine metabolism
	ko00983	Drug metabolism - other enzymes
	ko01212	Fatty acid metabolism
	ko02010	ABC transporters
	ko00232	Caffeine metabolism
B-228-L	ko04979	Cholesterol metabolism
	ko03008	Ribosome biogenesis in eukaryotes
	ko00260	Glycine, serine and threonine metabolism
	ko00920	Sulfur metabolism
	ko00670	One carbon pool by folate
	ko05410	Hypertrophic cardiomyopathy
	ko05414	Dilated cardiomyopathy
	ko03420	Nucleotide excision repair
	ko00130	Ubiquinone and other terpenoid-quinone biosynthesis
	ko00520	Amino sugar and nucleotide sugar metabolism

Supplementary Table S10. The GO enrichment analysis of common DEGs across groups.

Time and expression profile	GO ID	Description	Class
96 hpi up-regulated	GO:0006457	protein folding	Biological Process
	GO:0044183	protein binding involved in protein folding	Molecular Function
	GO:0007316	pole plasm RNA localization	Biological Process
	GO:0019094	pole plasm mRNA localization	Biological Process

	GO:0017038	protein import	Biological Process
	GO:0060811	intracellular mRNA localization involved in anterior/posterior axis specification	Biological Process
	GO:0060810	intracellular mRNA localization involved in pattern specification process	Biological Process
	GO:0050479	glyceryl-ether monooxygenase activity	Molecular Function
	GO:0051087	chaperone binding	Molecular Function
	GO:0010035	response to inorganic substance	Biological Process
96 hpi down-regulated	GO:0071942	XPC complex	Cellular Component
	GO:0000111	nucleotide-excision repair factor 2 complex	Cellular Component
	GO:1990165	single-strand break-containing DNA binding	Molecular Function
	GO:0031151	histone methyltransferase activity (H3-K79 specific)	Molecular Function
	GO:0000404	heteroduplex DNA loop binding	Molecular Function
	GO:0000405	bubble DNA binding	Molecular Function
	GO:0032135	DNA insertion or deletion binding	Molecular Function
	GO:0003684	damaged DNA binding	Molecular Function
	GO:0010777	meiotic mismatch repair involved in reciprocal meiotic recombination	Biological Process
	GO:0004578	chitobiosyldiphosphodolichol beta-mannosyltransferase activity	Molecular Function
228 hpi up-regulated	GO:0050479	glyceryl-ether monooxygenase activity	Molecular Function
	GO:0016714	oxidoreductase activity, acting on paired donors, with incorporation or reduction of molecular oxygen, reduced pteridine as one donor, and incorporation of one atom of oxygen	Molecular Function
	GO:0008061	chitin binding	Molecular Function
	GO:0004497	monooxygenase activity	Molecular Function
	GO:0005506	iron ion binding	Molecular Function
	GO:0016040	glutamate synthase (NADH) activity	Molecular Function
	GO:0046485	ether lipid metabolic process	Biological Process
	GO:0006662	glycerol ether metabolic process	Biological Process
	GO:0018904	ether metabolic process	Biological Process
	GO:0016705	oxidoreductase activity, acting on paired donors, with incorporation or reduction of molecular oxygen	Molecular Function
228 hpi down-regulated	GO:0010917	negative regulation of mitochondrial membrane potential	Biological Process
	GO:0045837	negative regulation of membrane potential	Biological Process
	GO:0035794	positive regulation of mitochondrial membrane permeability	Biological Process
	GO:0046902	regulation of mitochondrial membrane permeability	Biological Process
	GO:0010940	positive regulation of necrotic cell death	Biological Process
	GO:0090559	regulation of membrane permeability	Biological Process
	GO:0010939	regulation of necrotic cell death	Biological Process
	GO:0070265	necrotic cell death	Biological Process
	GO:0051881	regulation of mitochondrial membrane potential	Biological Process
	GO:0035578	azurophil granule lumen	Cellular Component

Supplementary Table S11. The KEGG enrichment analysis of common DEGs across groups.

Time and expression profile	Pathway ID	Description
96 hpi up-regulated	ko04141	Protein processing in endoplasmic reticulum
	ko05205	Proteoglycans in cancer
	ko04210	Apoptosis
	ko03060	Protein export

	ko05217	Basal cell carcinoma
	ko04145	Phagosome
	ko00500	Starch and sucrose metabolism
	ko03410	Base excision repair
	ko04340	Hedgehog signaling pathway
	ko04612	Antigen processing and presentation
96 hpi down-regulated	ko03420	Nucleotide excision repair
	ko01040	Biosynthesis of unsaturated fatty acids
	ko00062	Fatty acid elongation
	ko00510	N-Glycan biosynthesis
	ko00310	Lysine degradation
	ko00513	Various types of N-glycan biosynthesis
	ko04146	Peroxisome
	ko04120	Ubiquitin mediated proteolysis
	ko04142	Lysosome
	ko01100	Metabolic pathways
228 hpi up-regulated	ko01523	Antifolate resistance
	ko00790	Folate biosynthesis
	ko00520	Amino sugar and nucleotide sugar metabolism
	ko01100	Metabolic pathways
228 hpi down-regulated	ko04979	Cholesterol metabolism
	ko04142	Lysosome
	ko05203	Viral carcinogenesis
	ko02010	ABC transporters
	ko03022	Basal transcription factors
	ko03420	Nucleotide excision repair
	ko04146	Peroxisome
